# The Association between 5HT2A T102C and Behavioral and Psychological Symptoms of Dementia in Alzheimer's Disease: A Meta-Analysis

**DOI:** 10.1155/2017/5320135

**Published:** 2017-11-16

**Authors:** Liang Tang, Yan Wang, Yiwei Chen, Lianghui Chen, Shui Zheng, Meihua Bao, Ju Xiang, Huaiqing Luo, Jianming Li, Yungui Li

**Affiliations:** ^1^Department of Human Anatomy, Histology and Embryology, Institute of Neuroscience, Changsha Medical University, Changsha, China; ^2^School of Basic Medical Science, Changsha Medical University, Changsha, China; ^3^Experiment Center for Function, Changsha Medical University, Changsha, China; ^4^Key Laboratory for Fertility Regulation and Birth Health of Minority Nationalities of Yunnan Province, Judicial Expertise Center, Yunnan Population and Family Planning Research Institute, Kunming, China; ^5^Department of Neurology, Xiangya Hospital, Central South University, Changsha City, Hunan Province, China; ^6^Department of Biological Science, Hunan Environment Biological Polytechnic, Hengyang, China

## Abstract

The serotonin receptor gene (5-HT2A) has been reported to be a susceptible factor in behavioral and psychological symptoms of dementia (BPSD) in Alzheimer's disease (AD). However, previous results were conflicting. We aim to investigate the association of 5-HT2A T102C with BPSD in AD using a meta-analysis. Studies were collected using PubMed, Web of Science, the Cochrane Library databases, Chinese National Knowledge Infrastructure (CNKI), and Embase. Pooled odds ratios (ORs) with 95% confidence intervals (CIs) were used to assess associations. Nine studies with 1899 AD patients with/without BPSD were included in this meta-analysis. The 102C and CC genotypes were associated with psychosis in AD (102C: *p* < 0.00001, OR [95% CI] = 3.19 [2.12–4.79]; CC: *p* < 0.00001, OR [95% CI] = 7.24 [3.60–14.59]). The TT genotype was significantly associated with hallucinations, aberrant motor behavior, and psychosis in AD (hallucinations: *p* = 0.001, OR [95% CI] = 0.52 [0.36–0.77]; aberrant motor behavior: *p* = 0.03, OR [95% CI] = 0.58 [0.35–0.95]; and psychosis: *p* = 0.002, OR [95% CI] = 0.34 [0.17–0.67]). No association was observed between T102C alleles or genotypes and delusions, agitation/aggression, depression, and apathy (*p* > 0.05). Thus, the 5HT2A T102C might be a susceptible factor for hallucinations, aberrant motor behavior, and psychosis in AD. The potential mechanism of this polymorphism in BPSD in AD requires further exploration.

## 1. Introduction

Cognitive decline is one of the major neuropsychiatric features in Alzheimer's disease (AD) [1]. However, a variety of other neuropsychiatric features, such as depression, delusions, hallucinations, aberrant motor behavior (AMB), and anxiety, known as the behavioral and psychological symptoms of dementia (BPSD), are also present [2]. The incidence of BPSD is not consistent in AD patients. To date, the aetiology for BPSD in AD is not clear yet. Studies have been proposed that these symptoms may be related to the loss of different neuronal populations, such as the parahippocampal gyrus and the dorsal raphe nucleus, specific neurotransmitters, including dopamine and serotonin, and genetic components [3–5].

Serotonin (5-hydroxytryptamine, 5-HT) is a key neurotransmitter involved in many aspects of human and animal behavior, including aggression, hallucinations, delusions, depression, anxious behavior, and the regulation of appetite [6–8]. The action of 5-HT is mediated by 5HT receptors, especially 5HT2A and 5HT2C, which have been previously examined as possible factors for susceptibility to certain aspects of BPSD and many other psychiatric diseases, such as bipolar affective disorder and schizophrenia [9–12]. Moreover, postmortem and biopsy studies have shown changes in the expression levels and receptor binding of 5-HT receptors in brains of AD patients [13]. Consequently, many studies have examined the relationship between several polymorphisms of serotonin genes, especially the 5HT2A gene, and psychotic symptoms in AD patients. Recent observations indicate that a silent mutation presenting at position 102 (T102C) in this receptor gene may be a risk factor for psychotic symptoms in the course of AD.

Holmes et al. [14] firstly reported the association between the 5-HT2A C102 polymorphism and the hallucinations in AD, which was subsequently confirmed by Nacmias et al. in an European population [15]. Rocchi et al. reported the significant association between 5-HT2A C102 and psychosis [16], which was also followed by Lam et al. in a Chinese cohort [17]. In addition, the 5HT2A 102C is also reported to be associated with schizophrenia [18, 19], agitation [14, 17], apathy [17], AMB [17], and depression [20] in AD. Similarly, the 5HT2A T102 was reported to be associated with delusions [21], agitation [21], and depression [20] in AD. However, Micheli et al. [22] proposed that 5HT2A C102T may not be involved in psychosis in AD. And no statistically significant differences in the distributions of allele and genotype frequencies were found between AD patients with and AD patients without psychotic symptoms by Scordo et al. [23] and Pritchard et al. [24].

Due to the conflicting findings and limited availability of sample numbers in some studies, we aim to investigate the genetic associations between 5HT2A C102T and BPSD in AD patients by a meta-analysis.

## 2. Materials and Methods

### 2.1. Literature Search

Two independent authors (Liang Tang and Jianming Li) searched the PubMed, Embase, Web of Science, the Cochrane Library databases, and Chinese National Knowledge Infrastructure (CNKI) databases within the published years before 31 February, 2017, on the association between 5HT2A polymorphism and BPSD in Alzheimer's disease. The following terms were used in searching: “5HT2A” or “neurotransmitter 5-hydroxytryptophan 2A Receptor” or “serotonin receptor 2A” or “Serotonin 2A Receptor” or “HTR2A” and “Alzheimer's disease” or “AD” and “behavioral and psychological symptoms of dementia” or “BPSD” and “psychological symptom” and “polymorphism” or “polymorphisms”. Meanwhile, other potentially relevant literature was identified by manual search of references of eligible studies. No language was restricted.

### 2.2. Eligibility Criteria

*Inclusion Criteria*. They were as follows: (1) The publication was an unrelated case-control study. (2) The study examines the association of 5HT2A T102C and psychological symptoms of AD. (3) The genotype in the control group satisfied the Hardy-Weinberg equilibrium (HWE). (4) The frequencies of alleles or genotypes in the case and control groups could be extracted.

*Exclusion Criteria*. They were as follows: (1) repeat studies; (2) abstracts, letters, reviews, or editorial articles; (3) publications that did not fit the inclusion criteria.

### 2.3. Data Extraction

Data from the identified studies were extracted independently by Yan Wang and Shui Zheng using a standardized extraction form. Any disagreements were resolved through discussion among the authors to achieve a consensus. The following information was recorded for each study: first author, year of publication, ethnicity, assessment, number of patients with/without psychological symptoms, types of BPSD, positive results in each study, number of alleles, and genotype.

### 2.4. Quality Assessment

The quality of individual studies was assessed independently by two reviewer (Fang Li and Ju Xiang) according to the Jadad scale [25]. Four items were assessed, including source of controls, specimens, sample size, and evidence of HWE. The quality scores ranged from 0 to 5 (0 being the lowest and 5 being the highest). Only studies with a score of 3 or higher were included.

### 2.5. Statistical Methods

The odds ratio (OR) and 95% confidence interval (95% CI) were calculated for evaluating the association between 5HT2A T102C and BPSD in AD risk using the RevMan 5 (Oxford, UK) and STATA12.0 (StataCorp, College Station, TX, USA). The pooled ORs were calculated using the C versus T, TT versus CT/CC, and CC versus TT/CT genetic models. The statistical significance of the OR was determined using the* Z* test. Statistical heterogeneity was tested using *χ*^2^-based* Q* test and the *I*^2^ statistic. When there was no significant heterogeneity across studies (*I*^2^ < 50%), the fixed effect model (Mantel–Haenszel method) was used for meta-analysis. Otherwise, the random effect model (the DerSimonian and Laird method) was used. Sources of heterogeneity were evaluated by stratification analysis, according to the study characteristics. Sensitivity analysis was performed to assess the stability of results. The publication bias was detected with Begg's test and Egger's test. *p* < 0.05 was considered statistically significant.

## 3. Results

### 3.1. Characteristics of Eligible Studies

**Figure 1:**
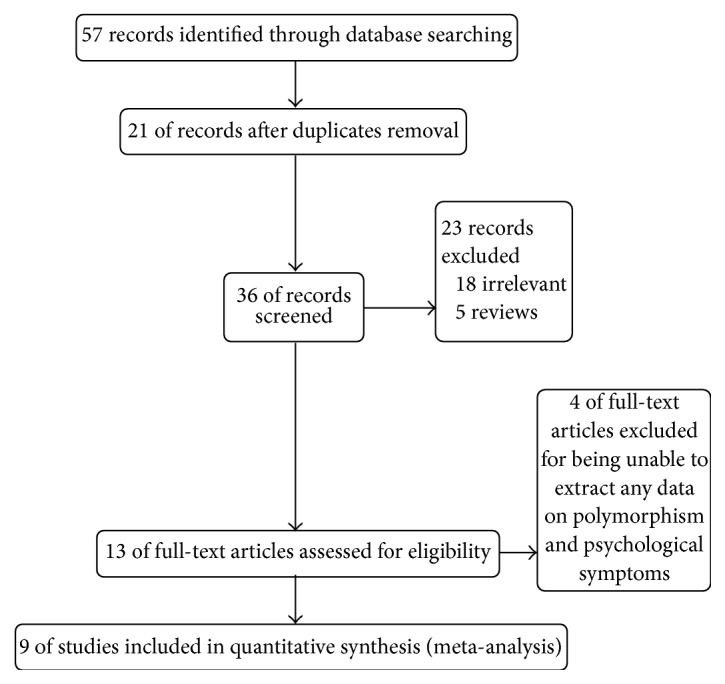
PRISMA flow chart of studies inclusion and exclusion.

The detailed steps of our literature search are shown in Figure 1. A total of 57 relevant articles were retrieved from various databases, of which 36 were included after scanning the titles; 21 were removed due to duplication, 18 for irrelevance, 5 for being reviews, and 3 for unavailable data related to the association between 5HT2A T102C and psychological symptoms of Alzheimer's disease and 1 was removed for non-case-control design. Finally, 9 studies [14–17, 20, 22, 24, 26, 27] meeting the criteria were retained for meta-analysis. The basic characteristics of enrolled patients are shown in Table 1.

### 3.2. Results of the Meta-Analysis

**Figure 2:**
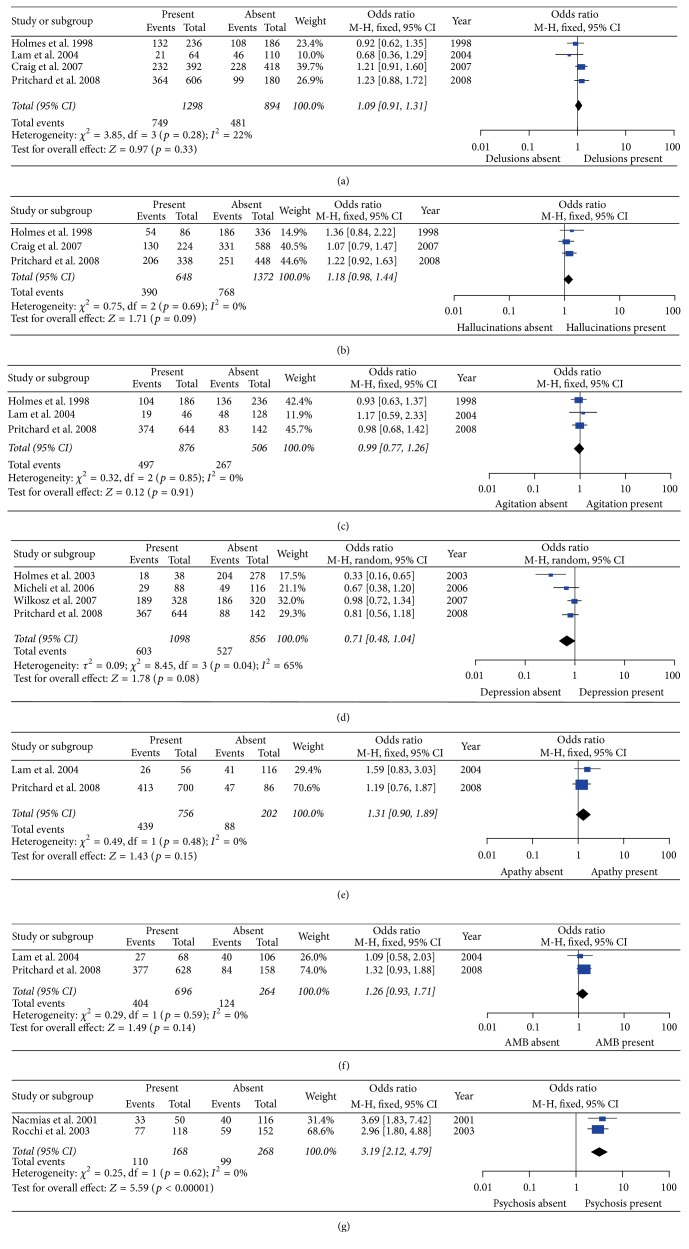
Forest plots of odds ratios for the association between 5HT2A C102T C versus T model and the risk of psychological symptoms of Alzheimer's disease. (a) Delusions; (b) hallucinations; (c) agitation; (d) depression; (e) apathy; (f) aberrant motor behavior (AMB); (g) psychosis.

Significantly increased risk for AD with psychosis (*p* < 0.00001, OR [95% CI] = 3.19 [2.12–4.79]) was found to be associated with 5HT2A C102 under the allelic model. No significant association was found between 5HT2A C102 and delusions, hallucinations, agitation/aggression, depression, apathy, and aberrant motor behavior susceptibility in the analysis as a whole (Table 2 and Figure 2).

**Figure 3:**
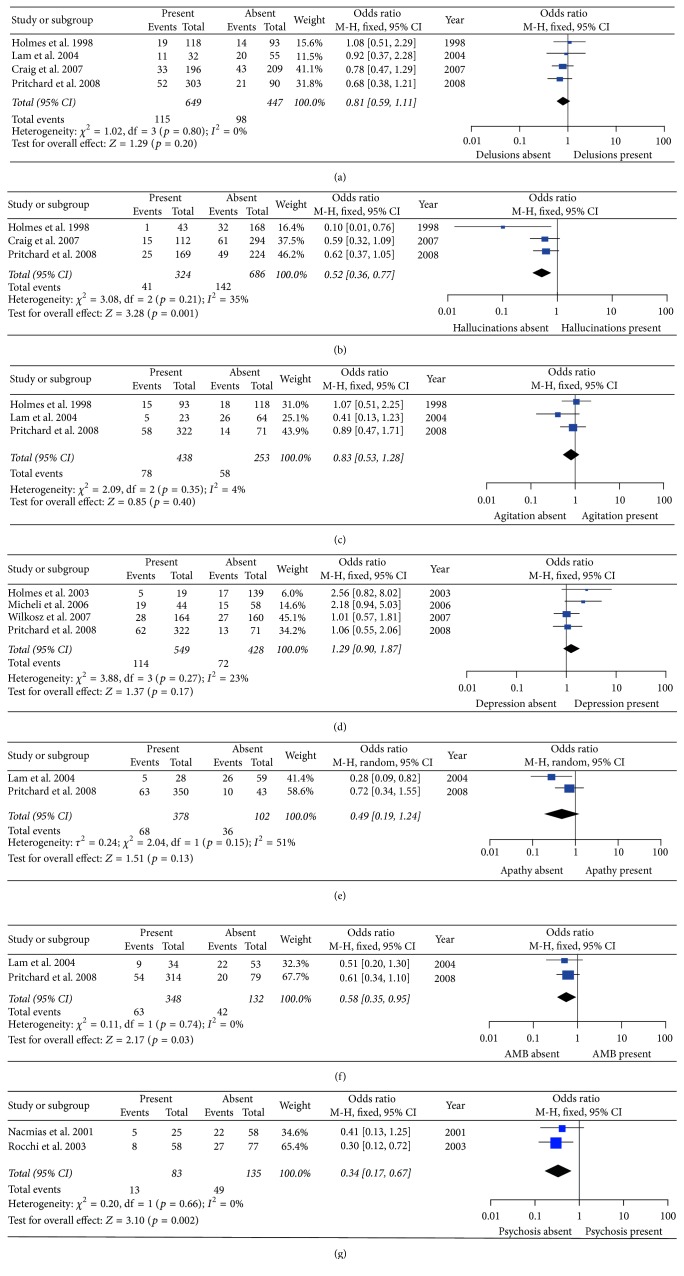
Forest plots of odds ratios for the association between 5HT2A C102T TT versus CT/CC model and the risk of psychological symptoms of Alzheimer's disease. (a) Delusions; (b) hallucinations; (c) agitation; (d) depression; (e) apathy; (f) aberrant motor behavior (AMB); (g) psychosis.

On the other hand, significant associations were found between 5HT2A T102C and hallucinations, aberrant motor behavior, and psychosis under the TT versus CT/CC model (hallucinations: *p* = 0.001, OR [95% CI] = 0.52 [0.36–0.77]; aberrant motor behavior: *p* = 0.03, OR [95% CI] = 0.58 [0.35–0.95]; and psychosis: *p* = 0.002, OR [95% CI] = 0.34 [0.17–0.67]). No association was observed between 5HT2A T102C and delusions, agitation/aggression, depression, and apathy susceptibility under the TT versus CT/CC model (Table 2 and Figure 3).

**Figure 4:**
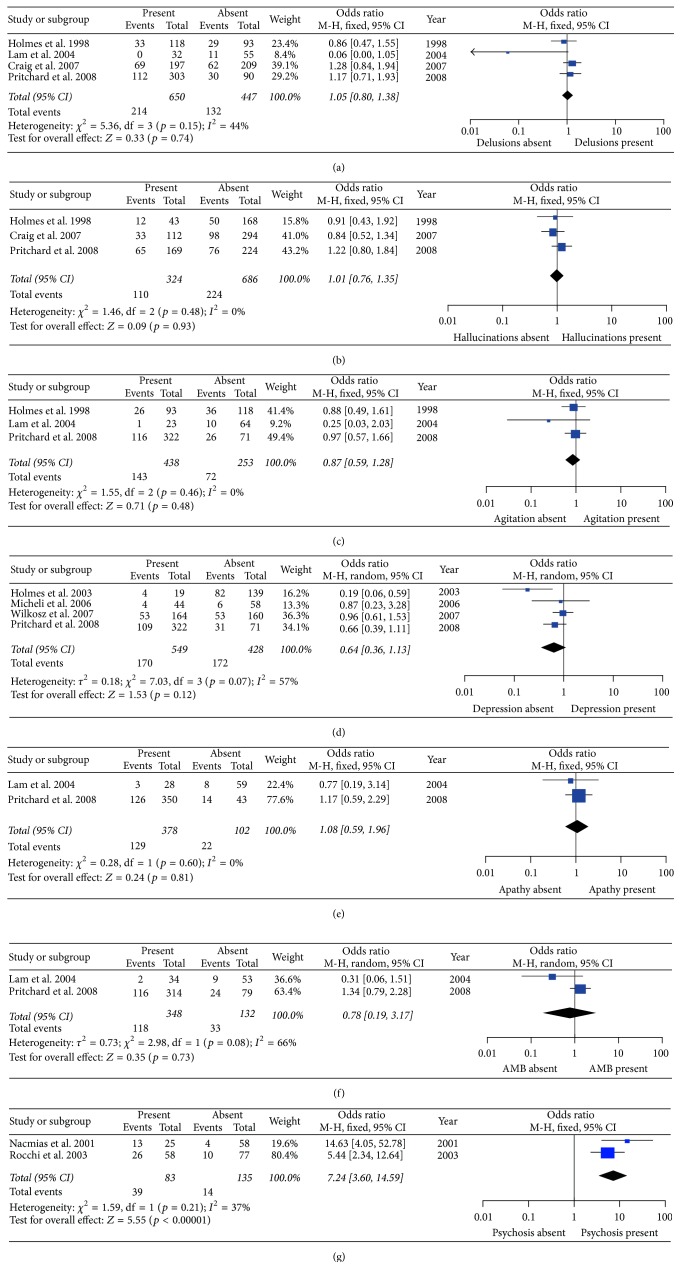
Forest plots of odds ratios for the association between 5HT2A C102T CC versus CT/TT model and the risk of psychological symptoms of Alzheimer's disease. (a) Delusions; (b) hallucinations; (c) agitation; (d) depression; (e) apathy; (f) aberrant motor behavior (AMB); (g) psychosis.

Furthermore, significant associations were confirmed between 5HT2A T102C and psychosis (*p* < 0.00001, OR [95% CI] = 7.24 [3.60–14.59]) under the CC versus TT/CT model. No other evident associations between 5HT2A T102C and delusions, hallucinations, agitation/aggression, depression, apathy, and aberrant motor behavior susceptibility under the CC versus TT/CT model were observed (Table 2 and Figure 4).

### 3.3. Sources of Heterogeneity

Significant heterogeneity was observed between 5HT2A 102C and depression (*I*^2^ = 65%, *p* = 0.04). This heterogeneity was contributed mainly by one positive study [20]. Removal of this study from meta-analysis gave 0% (*p* = 0.48) heterogeneity and showed that it had the highest effect on 5HT2A T102C allelic association with the effect of depression in AD.

For delusions, hallucinations, agitation/aggression, apathy, aberrant motor behavior, and psychosis, no significant heterogeneity was detected among all studies under the allelic model, TT versus CT/CC model, and CC versus TT/CT model (*p* > 0.05) (Figures 2, 3, and 4 and Table 2).

### 3.4. Sensitivity Analysis

**Figure 5:**
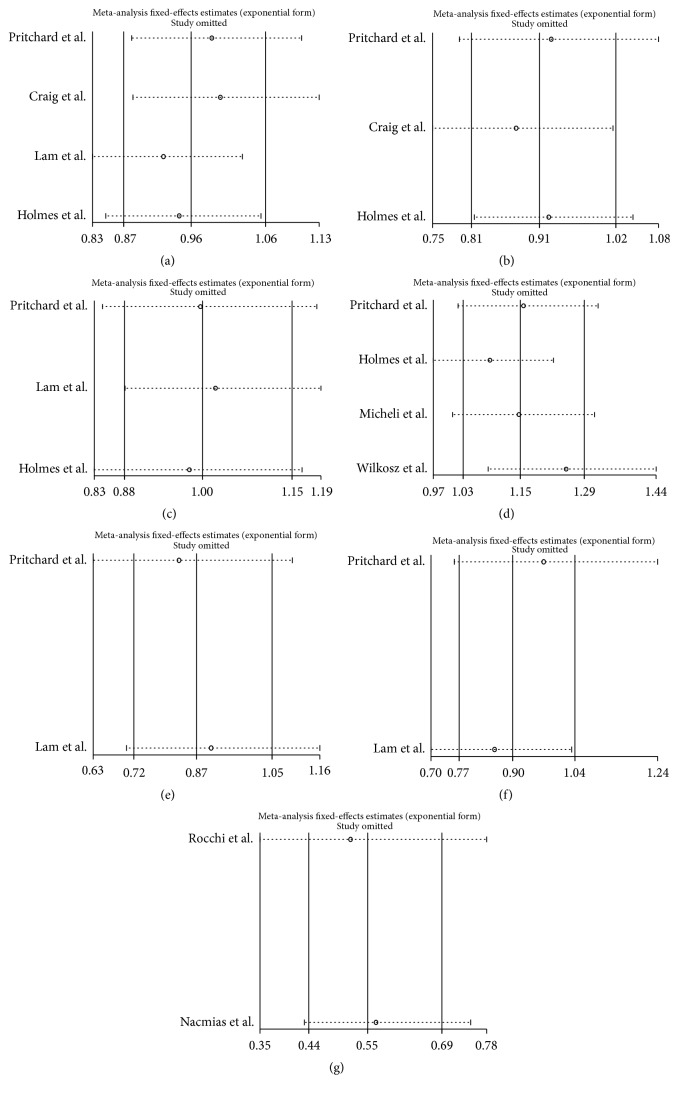
The influence of each study by removal of individual study for allelic model. (a) Delusions; (b) hallucinations; (c) agitation; (d) depression; (e) apathy; (f) aberrant motor behavior (AMB); (g) psychosis.

A sensitivity analysis that excluded the influence of a single study on the overall risk estimate by excluding one study at a time was confirmed. The ORs were not significantly altered in the allelic model (Figure 5).

### 3.5. Publication Bias

**Figure 6:**
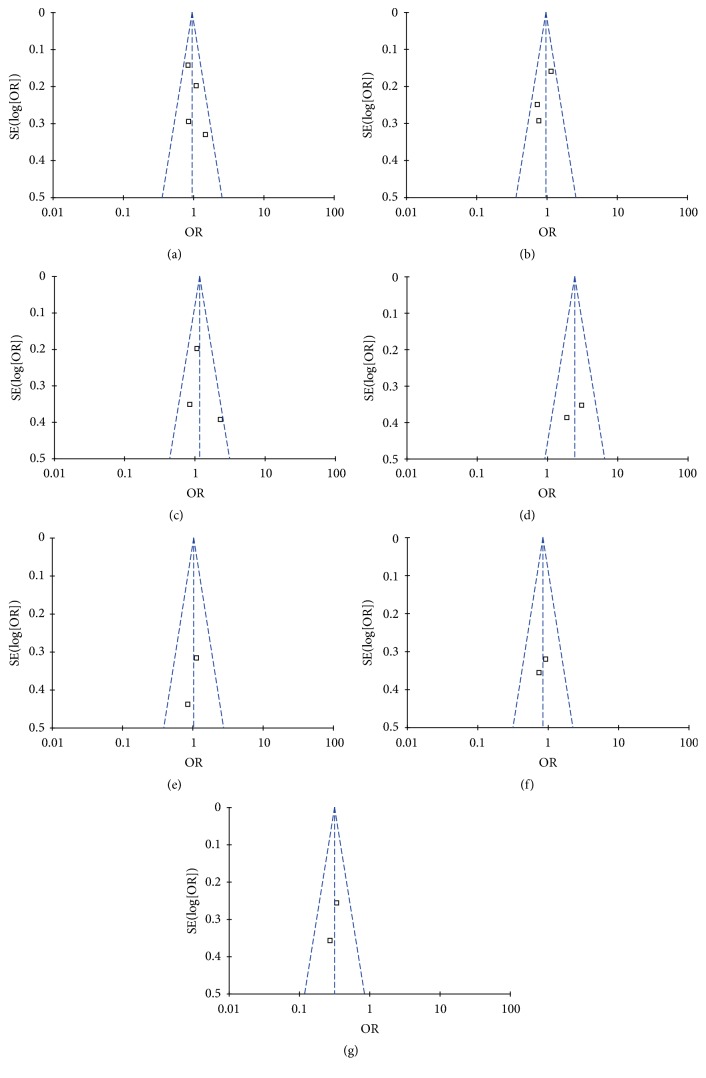
Funnel plot of publication bias for the association between 5HT2A C102T and the risk of psychological symptoms of Alzheimer's disease. (a) Delusions; (b) hallucinations; (c) agitation; (d) depression; (e) apathy; (f) aberrant motor behavior (AMB); (g) psychosis.

Begg's test and Egger's test were used to evaluate publication bias. The *p* value for Egger's linear regression test is shown in Table 3. Begg's test and Egger's test were not used in apathy, aberrant motor behavior, and psychosis due to a lack of sufficient data. No obvious publication bias was observed for delusions, hallucinations, or agitation/aggression (*p* > 0.05). The shape of funnel plot did not reveal any obvious asymmetry (Figure 6).

## 4. Discussion

This meta-analysis investigated the association between 5HT2A C102T and psychological symptoms in AD. The results demonstrated that the C allele and CC genotype of 5HT2A C102T were likely to be associated with psychosis in AD. The TT genotype of 5HT2A C102T was associated with hallucinations, AMB, and psychosis in AD.

5-HT and its receptors, particularly the 5-HT2A receptor, are considered to play a potential role in cognitive behaviors and psychiatric conditions such as depression, schizophrenia, and AD, as suggested by a large amount of pharmacological and neurobiological evidence [13, 28–31]. Moreover, decreases in density and specific binding of the 5HT2A receptor in the frontal and temporal cortex, hippocampus, and amygdala have been identified in AD patients [32–34]. Another study suggests that the presence of prominent behavioral problems, including depression and aggressive behavior, is also associated with 5-HT2A receptor losses [35].

Many studies have examined the relationship between polymorphisms of the 5HT2A gene and AD, as well as BPSD in AD patients. The mechanism by which 5-HT2A C102T alters the action of 5-HT in synaptic transmission remains unknown. Recent studies have shown that the TT genotype of 5HT2A C102T seems to be associated with higher platelet [36] and brain [19] 5-HT2A receptor density, which indicated an increased susceptibility for delusion symptoms in AD patients. In AD, both the 102T and 102C alleles have been linked to psychotic symptoms. Because the polymorphism was a synonymous change, most studies hypothesize that 5HT2A C102T polymorphism might be in linkage disequilibrium with other functional polymorphism(s) that may regulate and, thus, influence receptor density. This may reflect the influence of a separate gene existing in linkage disequilibrium. Notable, 5HT2A C102T polymorphism has been shown to be in linkage disequilibrium with the G1438A polymorphism in the promoter of the same gene, which could affect the expression levels of the 5HT2A receptor protein [37].

The underlying mechanism for 5-HT2A T102C in psychosis in AD is not well understood. It was hypothesized that increased frequency of the 5-HT2A 102C allele in APP-linked families may have further relevance in APP processing and then the BPSD in AD [38]. Two out of nine association studies have investigated the 5HT2A T102C polymorphism with psychosis and found an increase in the C allele or CC genotype in AD with psychosis [15, 16]. A significant association was also observed between the 5HT2A C102 allele and CC genotype and psychosis in AD in our meta-analysis study, which confirms that genetic variation at the T102C locus is associated with prominent psychotic features of psychosis in AD and that the 102C allele could play an important role in the clinical course of late-onset AD. Thus, the 5HT2A C102 allele and CC genotype were risk factors in BPSD of psychosis in AD and seemed to be reliable for the higher statistic power compared to that in the previous studies with moderate sample size.

Three studies conducted the genetic association between 5-HT2A C102T and hallucinations [14, 24, 26]. Holmes et al. have reported a significant association between the 5-HT2A C102 allele and the presence of hallucinations (auditory and visual hallucination) in a British population [14]. However, this positive result was not replicated in other British populations [24, 26]. And AD patients who are heterozygous for 5-HT2A T102C are more likely to hallucinate compared to homozygotes [14]. However, we found that the homozygotes (TT) are more likely to hallucinate compared to homozygous CC and heterozygous CT in AD. This contradictory finding is not easy to explain, and the inconsistent results might be due to relatively small sample sizes. Another possibility for the failure to replicate positive results could be differences in diagnostic criteria and genetic heterogeneity.

Only two previous researches reported the association between 5HT2A C102T and AMB [17, 24]. Lam et al. observed a statistically significant increase in the CC genotype in the presence of AMB [17]. However, negative results were found by Pritchard et al. [24]. We observed a significant increase in the TT genotype, but not the CC or CT genotype, in the presence of AMB in this meta-analysis. The function of 5HT2A C102T in AMB is not clear. Evidence of a significant loss of 5-HT2A receptor was reported in both postmortem and in vivo studies on AD patients with prominent behavioral symptoms [20]. Moreover, selective 5HT2A antagonists inhibit the head shake and twitch induced by 5HT2A agonists in rat models, which may suggest a role of this receptor gene in the pathology of AMB [39].

We noticed that Ramanathan and Glatt [40] have conducted a meta-analysis on the association between the 5HT2A C102T and BPSDs including psychosis, delusions, and hallucinations. And significant association was only found between the 5HT2A C102 and psychosis, but not delusions, and hallucinations. Our meta-analysis included three more studies (study conducted by Assal et al. [21] was excluded for non-case-control design) with three more BPSDs (agitation/aggression, apathy, and aberrant motor behavior) and suggested a significant association between TT genotype and hallucinations in AD patients.

Limitations should be mentioned. Firstly, the number of patients was relatively small and may influence the outcomes. Only a total of nine studies were included in the present meta-analysis. Among them, 4, 3, 3, 4, 2, 2, and 2 studies are related to delusions (749 cases and 481 controls), hallucinations (390 cases and 768 controls), agitation/aggression (497 cases and 267 controls), depression (603 cases and 537 controls), apathy (439 cases and 88 controls), aberrant motor behavior (404 cases and 124 controls), and psychosis (110 cases and 99 controls), separately. Secondly, AD is a multifactorial disease. Gene-gene interactions may play important roles in the pathology of BPSD in AD, but most studies lack information about gene-gene interactions. Thirdly, most of the patients in the present study were Caucasians, which may limit the general application of the results to other populations.

## 5. Conclusions

The current meta-analysis suggests an increased risk of psychological symptoms of psychosis in AD for the 5HT2A C102 allele and CC genotype and a decreased risk of hallucinations, aberrant motor behavior, and psychosis in AD for the 5HT2A TT genotype. To confirm these results, further study with larger sample size and multiple ethnicities is necessary.

## Figures and Tables

**Table 1:** Characteristics of eligible studies included in the meta-analysis.

Author (year)	Ethnicity	Assessment	Number of patients	BPSD	Positive results	Quality assessment
Pritchard et al. 2008	British	NPI	393	Delusions, hallucinations, agitation, depression, apathy, and AMB	Increased C allele and CC genotype with hallucinations, delusions, psychosis, and aberrant motor behavior (*p* < 0.05)	5

Craig et al. 2007	British	NPI	406	Delusions, hallucinations	No significant association was found	5

Lam et al. 2004	Chinese	NPI	87	Delusions, agitation, apathy, and AMB	Increased CC genotype with delusions (*p* = 0.02), agitation (*p* = 0.04), apathy (*p* = 0.03), and AMB (*p* = 0.05)	3

Holmes et al. 2003	British	CAMDEX	158	Depression	Increased TT and CC genotype with depression (*p* = 0.007)	4

Holmes et al. 1998	British	CAMDEX/MOUSEPAD	211	Delusions, hallucinations, and agitation	Increased C allele with hallucinations (*p* < 0.05)	5

Rocchi et al. 2003	Italian	NPI	135	Psychosis	Increased CC genotype (*p* < 0.001) with psychosis	3

Micheli et al. 2006	Italian	NPI/MMSE	208	Depression	No significant association was found	4

Nacmias et al. 2001	Italian	Semistructured interview	83	Psychosis	Increased CC genotype (*p* < 0.0001) and C allele (*p* < 0.0001) with psychosis	3

Wilkosz et al. 2007	American	DSM-IV	324	Depression	No significant association was found	5

BPSD: behavioral and psychological symptoms of dementia; AMB: aberrant motor behavior; NPI: neuropsychiatric inventory; CAMDEX: Cambridge Examination for Mental Disorders of the Elderly; MMSE: Mini-Mental State Examination; MOUSEPAD: Manchester and Oxford Universities Scale for the Psychological Assessment of Dementia.

**Table 2:** Pooled ORs and 95% CIs of the association between 5HT2A T102C and psychological symptoms of Alzheimer's disease.

Genetic Model	psychological symptoms	Number of studies	Test of association		Model	Test of heterogeneity	
			OR [95% CI]	*p* value		*p* value	*I* ^2^ (%)
C versus T	Delusions	4	1.09 [0.91–1.31]	0.33	F	0.28	22%
	Hallucinations	3	1.18 [0.98–1.44]	0.09	F	0.69	0%
	Agitation/aggression	3	0.99 [0.77–1.26]	0.91	F	0.85	0%
	Depression	4	0.71 [0.48–1.04]	0.08	R	0.04	65%
	Apathy	2	1.31 [0.90–1.89]	0.15	F	0.48	0%
	Aberrant motor behaviour	2	1.26 [0.93–1.71]	0.14	F	0.59	0%
	Psychosis	2	3.19 [2.12–4.79]	<0.00001	F	0.62	0%

TT versus CT/CC	Delusions	4	0.81 [0.59–1.11]	0.20	F	0.80	0%
	Hallucinations	3	0.52 [0.36–0.77]	0.001	F	0.21	35%
	Agitation/aggression	3	0.83 [0.53–1.28]	0.40	F	0.35	4%
	Depression	4	1.29 [0.90–1.87]	0.17	F	0.27	23%
	Apathy	2	0.49 [0.19–1.24]	0.13	R	0.15	51%
	Aberrant motor behaviour	2	0.58 [0.35–0.95]	0.03	F	0.74	0%
	Psychosis	2	0.34 [0.17–0.67]	0.002	F	0.66	0%

CC versus TT/CT	Delusions	4	1.05 [0.80–1.38]	0.74	F	0.15	44%
	Hallucinations	3	1.01 [0.76–1.35]	0.93	F	0.48	0%
	Agitation/aggression	3	0.87 [0.59–1.28]	0.48	F	0.46	0%
	Depression	4	0.64 [0.36–1.13]	0.12	R	0.07	57%
	Apathy	2	1.08 [0.59–1.96]	0.81	F	0.60	0%
	Aberrant motor behaviour	2	0.78 [0.19–3.17]	0.73	R	0.08	66%
	Psychosis	2	7.24 [3.60–14.59]	<0.00001	F	0.21	37%

F: fixed model; R: random model; OR: odd ratio; CI: confidence interval.

**Table 3:** Egger's linear regression test for funnel plot asymmetries of 5HT2A T102C.

Groups	Delusions	Hallucinations	Agitation	Depression	Apathy^a^	AMB^a^	Psychosis
*p* value	0.638	0.185	0.442	0.254	—	—	—

^a^Egger's linear regression test was cancelled in apathy, aberrant motor behavior, and psychosis for lack of sufficient data.
